# Automatic Passenger Counting on the Edge via Unsupervised Clustering

**DOI:** 10.3390/s23115210

**Published:** 2023-05-30

**Authors:** Giorgio Delzanno, Luca Caputo, Daniele D’Agostino, Daniele Grosso, Abdul Hannan Mustajab, Luca Bixio, Matteo Rulli

**Affiliations:** 1 DIBRIS, University of Genoa, via Dodecaneso, 35, 16146 Genoa, Italy; caputoluca48@gmail.com (L.C.);; 2 DIFI, University of Genoa, via Dodecaneso, 33, 16146 Genoa, Italy; 3 Flairbit S.r.l., via Maragliano, 6/5, 16121 Genoa, Italy

**Keywords:** IoT, WSN, edge computing, artificial intelligence

## Abstract

We present a device- and network-based solution for automatic passnger counting that operates on the edge in real time. The proposed solution consists of a low-cost WiFi scanner device equipped with custom algorithms for dealing with MAC address randomization. Our low-cost scanner is able to capture and analyze 802.11 probe requests emitted by passengers’ devices such as laptops, smartphones, and tablets. The device is configured with a Python data-processing pipeline that combines data coming from different types of sensors and processes them on the fly. For the analysis task, we have devised a lightweight version of the DBSCAN algorithm. Our software artifact is designed in a modular way in order to accommodate possible extensions of the pipeline, e.g., either additional filters or data sources. Furthermore, we exploit multi-threading and multi-processing for speeding up the entire computation. The proposed solution has been tested with different types of mobile devices, obtaining promising experimental results. In this paper, we present the key ingredients of our edge computing solution.

## 1. Introduction

### 1.1. Background, Motivations and Challenges

Currently, data coming from automatic passenger counting (APC) systems are a source of knowledge for solving a wide variety of urban transport issues, ranging from strategic reasoning on possible improvements of public services to real-time responses to critical traffic situations. These types of data are becoming a standard for developing concepts related to smart cities both for transport companies and authorities. Due to the increasing spread of low-cost video cameras and sensors, new technologies have been introduced to complement traditional methods based on manual counting, interviews and questionnaires; see e.g., [1,2,3].

According to the taxonomy presented in [1], in this paper, we are particularly interested in device- and network-based solutions in order to exploit passengers’ smartphones as signal emitters at the cost of designing customized receiving stations able to produce crowd density estimations. In this class of APC systems, there exist solutions based on wireless network signal processing, e.g., detection of WiFi or Bluetooth beacon messages emitted by passengers’ devices. For instance, it is well known that Bluetooth scanners can be used to compute a rough estimation of the number of nearby Bluetooth devices (smartphones, headphones, smart watches, etc.). WiFi scanners represent, at least in principle, a more robust and accurate method. A WiFi scanner can detect (2.4 and 5.8 GHz) signals in a range compatible with the size of a bus. Furthermore, WiFi probe messages, used for instance in the 802.11 WiFi protocol, include the sender MAC address in clear. At least in principle, they could be used for a precise counting of nearby devices (and therefore passengers). However, since 2017, iOS producers such as Apple and Android have started introducing the use of fake MAC addresses to reduce privacy violations. Fake addresses are generated via randomization algorithms in every procedure that requires some form of initial handshake or broadcasting in clear. Real MAC addresses are exchanged only after a communication channel has been established. The use of fake MAC addresses currently represents a main challenge for automatic passenger counting based on WiFi scanning [4,5,6,7,8,9,10].

### 1.2. Research Question

Our research question is whether, in the presence of fake MAC addresses, an 802.11 WiFi probe scanner could still be used for designing an effective, real-time automatic passenger counting procedure operating on the edge, i.e., without the need for transmitting collected data for off-line processing to an external server, therefore preserving data privacy.

### 1.3. Our Contribution

Our technical contribution can be divided into three main parts.

- We first designed a low-cost WiFi scanner able to analyze and process 802.11 probe requests emitted by passengers’ devices (smartphones, tablets, etc.). Our scanner consists of a Raspberry Pi 4 equipped with a SIM7600X 4G hat and an external BrosTrend AC650 WiFi adapter [11]. The 4G hat is used for LTE communications and for acquiring GPS positions in real time. The AC650 adapter is used both to achieve wide-area reception and to create a wireless network interface dedicated to packet sniffing. In our prototype, we considered 802.11 WiFi probe messages [12,13] collected via the tcpdump open source library [14]. These types of messages contain probe requests, i.e., message frames that contain the MAC address of the sender device. Our scanner captures 802.11 WiFi probe requests observed in a given time window and stored them locally in batches, for further processing.
- We designed a processing pipeline capable of combining data coming from different types of sensors (WiFi probe requests, GPS data, etc.), processing them on the fly using unsupervised clustering, namely a lightweight version of the DBSCAN algorithm [15], named DBSCAN${}_{L}$, and sending different types of aggregate and anonymized data to a cloud endpoint via the 4G SIM7600X hat.
- DBSCAN is a well-known method for unsupervised clustering. The main feature of this iterative algorithm is that it does not require to specify a priori the number of clusters used to partition elements. Clusters are dynamically adjusted via metrics used to evaluate the sparsity of the sets in the current partitioning.
- The DBSCAN${}_{L}$ algorithm is a simplified version of the DBSCAN algorithm. Our algorithm partitions an initial list of addresses according to two different features, namely RSSI and data rates, with an additional step based on other possible clustering criteria. Differently from the original version of DBSCAN, our algorithm only labels noise points, therefore maintaining labels assigned in previous steps. In other words, it does not merge overlapping clusters as in DBSCAN. This strategy seems to give better results in the considered case study.
- Concerning prototype implementation, the Python code that implements the algorithm is designed in a modular way in order to accommodate possible extensions of the pipeline, e.g., additional filters, data sources (e.g., Bluetooth data), and processing algorithms. Each module runs in a separate Python thread (e.g., communication tasks and data processing). To reduce overhead, Linux pipes are used to pass data between different stages of the pipeline (i.e., the standard output of a module is redirected to the standard input of the next module via a pipe channel). Threading is applied here in order to create producer–consumer pipelines within the same program, share the same address space, and thus gain in efficiency. Multiprocessing is used to define the Python (parallel) pipeline that implements the different steps required by the algorithm (probe sniffing, data preparation, estimate generation, and data distribution). At boot time, a graceful initialization process of the entire pipeline is used in order to take into consideration possible delays due to the GPS receiver and the 4G SIM7600 module cold-start procedure.
- We tested our edge device to collect benchmarks and datasets in indoor scenarios using different types of mobile devices including Android smartphones, iPhones and tablets. In the experiments in controlled scenarios, e.g., with a fleet of devices known in advance, the prototype returned estimation with 75–80% precision.

In addition to the technical contribution, we obtained promising results not only for what concerns crowd estimations in real scenarios but also useful information, in a sort of reverse engineering process, on the different randomization procedures (frequency of probe requests, smartphone status in which beacons are generated, shape of fake MAC addresses, etc.) adopted in different types of operating systems. These additional data could be applied for further refinements of the proposed solution in controlled scenarios (e.g., for monitoring a fleet of devices in emergency operations, etc.) and, more in general, for future extension of the proposed algorithms.

### 1.4. Originality and Reproducibility

This paper includes original work carried out during a collaboration between the University of Genoa and Flairbit S.r.l.. The source code of the entire system can be requested by contacting the authors.

### 1.5. Plan of the Paper

In Section 2, we discuss related work with a particular focus on non-image-based approaches. In Section 3, we summarize the main concepts underlying the 802.11 probe handshaking protocol and all issues related to MAC address randomization. In Section 4, we present the physical components of our edge device, together with some details on its configuration. In Section 5, we present the pipeline used for device detection. In Section 6, we present the software architecture used in the pipeline. In Section 7, we discuss the experimental results obtained with different types of smartphones and tablets in a variety of supervised and unsupervised scenarios. Finally, in Section 8, we address some conclusions and discuss future directions for our work.

## 2. Related Work

In this section, we discuss related work mainly focusing on wireless network signal processing techniques, e.g., detection of WiFi or Bluetooth beacon messages emitted by passengers’ devices. In [1], the authors present a survey of non-image-based approaches classified into device-free, namely RF-based and sensor-based (i.e., they do not expect participants to carry additional devices), and device-based, namely acoustic-based and network-based.

We focus the discussion of related work on device- and network-based approaches. In [16], the authors present a fixed system for crowd density estimations based on WiFi probe requests. Sentinel  [17] is a system that estimates occupancy in commercial buildings based on existing WiFi infrastructures. WiPin [18] is a system equipped with a front-end module on the WiFi router for occupancy estimation performed in real time, which also takes into account historical data in a back-end module. In [2], the authors proposed to use a mathematical model in conjunction with MAC addresses extracted from WiFi probe messages. They applied Kalman Filters to fit the considered model in order to provide estimations even in cases of missing historical data. The above-mentioned approaches do not take into consideration the additional noise due to MAC address randomization, a real challenge for using WiFi probe messages generated by smartphones [9].

In [19], the authors proposed a de-randomization algorithm to compute the probability that two random MAC addresses correspond to the same device. More precisely, they computed a score for each pair of random MAC addresses identified based on the timestamp and the frame sequence number (a 12 bit code in the Seq-ctl field). In [10], the author generates fingerprints of probe requests sent by a single device using information elements, inter-burst times and classification procedures based on the KNN algorithm. The results showed an accuracy of 75%. Inter-frame time has been considered in other approaches such as in [20].

In [4,5,6], the authors extend the above-mentioned approaches by considering context-dependent information contained in probe requests in a adaptive way, e.g., by considering the length of information elements as a further parameter for their clustering algorithm.

Some works merge static and dynamic information for improving the accuracy of the estimation. In [21], the authors developed a statistical model used to predict the number of on-board passengers traveling on buses based on manual passenger counts and logging of wireless data frames. The results are very interesting, but they are based on a ground truth of the model represented by the manual count of passengers and the fact that in 2017, only a few Android smartphones used randomization techniques.

In the same applicative context, the work of  [3] faces the problem of classifying MAC addresses of passengers and pedestrians by collecting probe messages with GPS information acquired by the scanner. This information is then coupled with static information as bus stop zoning and bus route circulation. While the results are interesting, a key drawback is that the authors assumed that each passenger carried only one WiFi device that can be uniquely associated with one passenger inside the bus.

In [7], an approach that can cope with commercial sensors as well as simple ad hoc scanning devices was proposed. This work represents an evolution of [19,22]. In [22], the authors aimed to infer flow densities and directions of transit of people on some areas (city streets or campuses), in particular with the goal of associating each device with the most likely path across a given set of predefined possibilities. However, this paper considers the use of real MAC address. This is the reason why it has been coupled with the de-randomized schema presented in [19] and an original ML-based technique for flow detection of people associated with real randomized MAC addresses.

In [8], the authors start from the considerations that most related works focus on either small supervised tests, e.g., campuses, offices or small, well-known city areas, or on extensive unsupervised tests in small cities. In the first case, the test bed is associated with reliable ground truth but suffers from a joint biased user base and typology. In the other case, instead, the tests lack the ground truth data to support classification. This is the reason why they propose a study based on data collected over a period of four years in five different location typologies (fair, football stadium, nightclub, plazas, and transport). The result is that the collected patterns mark different trends for each location, and therefore, models need to be tuned for each case of location.

## 3. 802.11 WiFi Protocol and MAC Address Randomization

In this section, we review the key elements of the 802.11 probe request protocol used in this work to identify distinct devices. According to the 802.11 WiFi protocol, mobile stations broadcast probe requests to discover 802.11 networks within their proximity. Probe requests advertise the mobile stations’ supported data rates and 802.11 capabilities. Access points (APs) receiving the probe request check to see if the mobile station has at least one common supported data rate. If they have compatible data rates, a probe response is sent advertising the SSID (wireless network name), supported data rates, encryption types if required, and other 802.11 capabilities of the AP.

### 3.1. 802.11 Probe Request Frames Structure

A probe request message frame is structured as follows. The frame header contains the following fields:

- Frame control, 2 bytes;
- Duration, 2 bytes;
- Destination Address (DA), 6 bytes;
- Source Address (SA), 6 bytes;
- BSSID, 6 bytes;
- Seq-ctl, 2 bytes.

The frame body contains the following fields:

- SSID and supported rates of variable length;
- FCS, 4 bytes.

Among the most important information is the SA (source address), which is the MAC address of the device requesting to establish the connection, and the SSID (service set identifier), which is used to specify the name of the network to connect to. Probe requests are actually sent in broadcast mode, i.e., received by all access points in the proximity of the device.

### 3.2. Device Detection via Probe Requests

**Figure 1:**
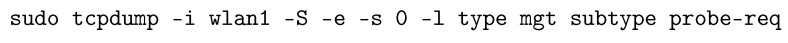
Use of tcpdump command for sniffing probe-requests.

Although all 802.11 frames exchanged between devices and access points can be captured and analyzed to obtain the estimated number of passengers, the idea of using only probe requests allows for a reduction in the number of data to be processed. This is important when considering that the system will have to process data arriving in real time. In addition, they are sent by clients only looking for a network to connect to, which leads to being able to focus on passenger devices and not on access points. Furthermore, probe requests are predominantly sent by devices that are not currently connected to any network when the device is in use. This is a common scenario for a transportation vehicle. Probe requests, being sent in the clear and therefore unencrypted, can be captured by a network traffic analysis tool, using an antenna tuned to the same frequency channel as the device that is transmitting the request. Our experiments were carried out mainly with tcpdump. The antenna used for packet capture must be set to monitor mode, so that all traffic on a particular channel can be listened to without being hooked to a particular network. The tcpdump tool can be used to filter and select 802.11 probe requests as in Figure 1.

**Figure 2:**
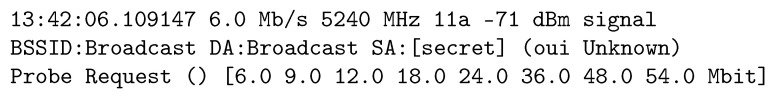
Record returned by tcpdump.

The option -i is used to specify the network interface in which listening and subsequent packet capture takes place. Using Linux terminology, wlan1 is specified, since the antenna used for capture, once installed and configured, will lock onto that interface. If the argument is not specified, listening takes place on the default network interface. The option -e is used to print the data link layer headers, while -s specifies how many bytes to capture per packet, thus the length in bytes of what to capture. The default value, indicated via -s 0, is 65535 bytes. The parameter type mgt subtype is used to specify the types of packets to capture, so that it filters all incoming packets by returning only those of a specific type. In this case, wanting to filter probe requests, we need to specify probe-req. An example of a record produced by tcpdump is shown in Figure 2.

The record contains information about the received probe request:

- A timestamp indicating when the probe request was captured, expressed in the format hh:mm:ss.frac;
- A number indicating the data rate supported at that particular time by the device, expressed in Mbits per second or Mb/s;
- The signal strength with which the message was received, expressed in signal dBm; tt will be seen, later, that it can be used to determine the distance of the device from the antenna;
- The BSSID, which is the MAC address of the access point on the network that the device is trying to contact if it was connected to it in the past, or broadcast if looking for new ones;
- The DA (destination address), the MAC address of the recipient of the request; it is usually broadcast so that all access points will see it;
- The MAC address of the device that is sending the request, i.e., the SA (source address);
- The type of frame being sent, in this case a probe request (); usually inside () is the SSID of the network the device is looking for if it is looking for one; otherwise, it remains empty;
- The list of data rates supported by the device sending the request that may be useful in the communication between the device and access point, indicated inside square brackets.

In the rest of the section, we focus our attention on the analysis of the following elements: MAC addresses and probe frequency, RSSI level, and supported data rates.

### 3.3. MAC Address Randomization

Until a few years ago, APC systems could count passengers on the basis of the MAC address alone, as each distinct address was potentially a passenger. This was because it is a physical address, used at the data link level, uniquely assigned to the device and inserted into the network card during production; thus, it is also used to uniquely identify the device globally.

A MAC address is composed of 48 bits divided into 6 octets of 8 bits, each representing a hexadecimal number; thus, they have the following structure and format: 00:00:00:00:00 to ff:ff:ff:ff:ff. The first three bytes are intended to represent the manufacturer of the device, whose numbers are assigned by the IEEE Registration Authority, and are named OUI (organizational unique identifier), while the second three bytes represent the network card (network interface controller), assigned by the manufacturer itself. It can be inferred from the above description that since these are unique addresses assigned to the device, they are potentially the best way to identify a passenger. However, to date, more and more devices implement MAC address randomization algorithms in order to avoid device tracking based on it and more specifically to ensure user privacy, which means that the probe requests will no longer contain the real MAC address, but rather one generated via software. Such addresses can be distinguished by the second byte, in that it is always equal to one of the following hexadecimal numbers: 2, 6, a, e. There is no unique way to implement randomization; each manufacturer implements it in its own way. For example, Apple began adding MAC address randomization starting with iOS 8, when devices are not associated with any WiFi network and with the screen off, while now it is also used when devices are connected to a network and with the screen on. Google, on the other hand, began implementing MAC address randomization starting with Android 8 when devices are looking for a network to connect to, while Android 9 included the ability to enable a developer option for a device to use a randomized MAC address when it is already connected to a network. The use of the randomized address while the device is already connected to a network, this time user-side, was instead included starting with Android 10, becoming the default setting in some manufacturers (e.g., Samsung). To evaluate the effective use of randomization, we examined a number of devices with different types of operating systems such as an Asus Zenfone 3 with Android 8 2016, a Samsung Galaxy M12 with Android 11 2021, and an Apple iPhone 6s with iOS 9 2015. Our experiments confirmed that iOS and Android operating system phones are, over time, increasingly switching to randomization mechanisms, making it clear that counting the MAC addresses alone in a distinct manner is not enough, as one risks having a much higher number of potential passengers than the real and actual number. Therefore, in the following sections, we explore probe requests with randomized MAC addresses and their contents, and we identify features to be used for classification tasks.

### 3.4. Frequency of Probe Requests

Devices that send probe requests using randomized MAC addresses tend to change MAC addresses frequently if they are not connected to any access point. It has been noted that there is a tendency for probe requests to be sent especially when the screen is on, which is useful considering that a large proportion of passengers use their smartphones during their trips, if only at certain times. In addition, the manufacturers themselves do not provide much information about how the randomization of MAC addresses takes place, and there is a possibility that without looping in all possible frequency channels when capturing probe requests, some information will be lost.

From a series of experimental results, we observed that the Samsung Galaxy M12 sends about three probe requests per minute, while the iPhone 6s seems to have a noisier trend, that is, an average of about 4.1 probe requests per minute, but starting from 7 captured addresses in the first minute and then dropping and flattening out to only 3 captured addresses. From here, it can be said that, apart from potential spikes, smartphones send probe requests with a certain number of distinct randomized MAC addresses over a period that fluctuates between 3 and 4 addresses per minute. Using frequency in this sense can be useful in order to associate a certain number of MAC addresses with a single device.

### 3.5. RSSI Data

RSSI, short for received signal strength indicator, is a measure that indicates how well one device can hear another during a communication. The unit of measurement is decibels on a logarithmic scale, i.e., dBm, which represents the signal strength captured in milliwatts. RSSI and dBm are two units of measurement that represent the same thing, namely the power of a signal. However, RSSI is a measurement designed to represent the relative quality of a given signal; thus, it is a relative value. The IEEE 802.11 standard states that RSSI is a value between 0 and 255, but each chip manufacturer can decide its maximum value within the number 255. The higher the value, the higher the signal strength. The dBm, on the other hand, is a logarithmic value that indicates the received signal power; thus, the closer it is to 0 dBm, the higher the power, and the farther away, the worse it becomes. Received signal power is inversely proportional to the distance between the access point and the device that requires connection with it. In Table 1, we show a set of probe requests with the first MAC address of the Samsung Galaxy M12 captured during a WiFi survey.

In Table 1, we show a set of probe requests with the first MAC address of the Samsung Galaxy M12 captured during a 269 WiFi survey. As can be seen, a device usually sends not just one probe request with the same MAC address, but a whole group, which usually starts at a lower RSSI value, then rises and finally returns. The highest value is the one to be considered, since it is the one that can be used as an estimate of the distance to the antenna. In the previous example, the value in question is −16 dBm, which indicates a good signal and therefore good proximity to the antenna. The question that arises is whether RSSI values can actually suffice to distinguish devices within a transport medium, as well as whether they are actually stable. The following are some results of a test in which we tried to capture probe requests with different MAC addresses from the same smartphone, but at different locations each time. For each new MAC address, only the probe request with the highest rate is reported.

The time series in Table 2 refers to a survey in which the device is less than one meter from the scanner.

The time series in Table 3 refers to a survey in which the device is more than four meters away from the scanner. In general, somewhat lower values are obtained, a sign that one has moved away from the antenna, but one still has a MAC address sent with −32 dBm, a value already found when one is much closer to the antenna, presented in the first time series. These data have been obtained by moving the device and by turning on and off the screen (to simulate a bus trip). More stable values have been observed in less dynamic scenarios. In general, using RSSI can be very useful to infer the hypothetical distance between the passenger and the antenna. At the same time, however, this value can be quite unstable and inaccurate, and thus if used alone, it may not be entirely effective.

### 3.6. Supported Data Rates

The last parameter for our experiments is the list of supported data (transfer) rates (or data rates), which are sent directly from the device, which is precisely why they are part of the 802.11 frame. They can be defined as the amount of data transferred over a connection within one second, and they cannot be greater than the bandwidth of the connection. They are also called bits per second or bit rate. Each device sends messages with the entire list containing all the data rates supported by the device, which is useful for future communications with the access point to which one will connect. Data rates can be used as an additional distinguishing element. For instance, Samsung Galaxy M12 supports higher speeds than the iPhone 6s, which is actually older. Therefore, when many probe requests are captured with very similar values for RSSI, data rates might be used to discover that they are sent from two or more different devices.

### 3.7. Other Similarity Measures

**Figure 3:**
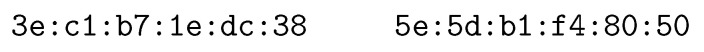
Comparison of MAC addresses.

In general, device manufacturers do not provide much information about how MAC address randomization is performed, except for Microsoft, which is known to use a hashing with the SHA algorithm in which it inserts some information inside, including the actual MAC address of the device and the SSID of the network to connect to, but it is very likely that other manufacturers also use some kind of hashing algorithm, which is very difficult to de-randomize. However, this does not exclude the possibility of finding any similarities between the MAC addresses. One idea that has been experimented with is to use a textual distance algorithm, e.g., Hamming distance, between the captured randomized MAC addresses. The Hamming distance identifies, within two strings of the same length, the number of positions at which the symbols are different. Consider for instance the MAC addresses in Figure 3.

Assuming that the separator “:” is not part of the string, the Hamming distance would be 10, since to be equal, out of 12 symbols, are only those in the second position and the one in the fifth position. Given that in general addresses are different from each other and it is rather difficult to find similar ones, the idea is to use textual similarity rather than distance, and in the above-mentioned example, the Hamming similarity would be 2. Given a set of collected MAC addresses, it is possible to use Hamming similarity to find addresses with the greatest number of the same symbols in the same locations within the same set, in order to reduce the number of addresses found to a number of devices consistent with the number present within the bus. At the same time, however, we want to understand “how many” symbols may be needed to classify MAC addresses as potentially part of the same device. We can take as an example the addresses coming from a Samsung Galaxy M12 in Table 4.

When trying to compare all the MAC addresses with each other, the maximum Hamming similarity that can be found within this list is three equal symbols. This table takes MAC addresses that have at least one correspondent with a similarity of three and groups them together as in Table 5.

The classes can be, for example, divided according to where intersections are present; thus, groups 1, 2, 3, 4 and 9 are part of the same class, as are 5 and 8, and finally 6 and 7, for a total of three possible classes, which could correspond to three possible devices within the list. In general, the more randomized MAC addresses are sent, the easier it is to find at least two with gradually increasing similarity as the MACs increase.

## 4. The WiFi Scanner Device

**Figure 4:**
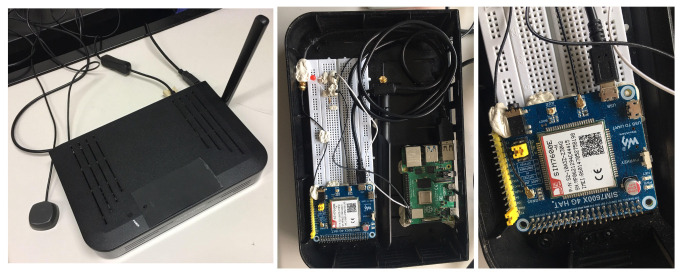
Prototype of the WiFi scanner with external BrosTrend AC650 WiFi antenna (**left**); the Raspberry Pi4 with LTE antenna and 4G hat (**center**); the SIM7600X 4G hat (**right**).

Our low-cost WiFi scanner is built on top of a Raspberry Pi4 Model B [23]. Pi4 Model B is equipped with the 64 bit quad-core Broadcom BCM2711 Cortex-A72 ARM v8 1.5 GHz processor and 4 Gb of RAM. It can be configured with the Raspberry Pi OS, an operating system based on Debian. Figure 4 illustrates the physical architecture of the scanner. The left picture shows the case containing the different modules and the external WiFi and GNSS antennas. The center picture shows the Raspberry Pi 4 computer connected, via a USB port, to a 4G SIM7600X hat and an LTE antenna. The right picture show the SIM7600X only.

The Waveshare SIM7600 4G HAT [24] is a 4G/3G/2G communication and GNSS positioning module, which supports LTE CAT4 up to 150 Mbps for downlink data transfer. It is based on the SIM7600X-H SIMCom 4G LTE Cat-4 Module [25]. The 4G HAT provides an RPi 40PIN GPIO extension header. It provides an onboard USB interface and a CP2102-USB-to-UART converter. The 4G hat is equipped with a SIM card slot and a TF card slot for storing data. More details on the hardware schematics are available on the manufacturers’ websites [24,25].

The SIM7600X 4G hat is connected via coaxial cables to the LTE and GNSS antennas. The 4G hat can be used to connect to the LTE network (2G/3G/4G) and to communicate over the Internet through standard protocols such as TCP/UDP and HTTP. It can also be used to obtain GPS coordinates in real time via an internal GNSS receiver. The 4G hat is equipped with USB ports, used to receive AT commands from the Pi4 computer, as well as a SIM card slot and LEDs to indicate its operation status.

A 1Nce sim card [26] is used for LTE communication. The 1Nce sim card, made specifically for IoT projects, provides 500 Mb of data volume, guaranteeing 1 Mb/s of data speed, and supports 3G and 4G cellular technologies. The connection to the 1nce mobile network service is implemented through the 1Nce APN, which assigns a private (class A) IP address to the device.

The internal WiFi antenna of the Pi4 Model B cannot be used in monitor mode, an operation mode needed for WiFi beacon detection. For this reason, we equipped the scanner with an external WiFi adapter. More specifically, we used the BrosTrend AC650 WiFi antenna compatible with the Raspberry OS. The adapter handles both 433 Mbps over 5 GHz band and 200 Mbps on 2.4 GHz WiFi signals and expands coverage by ensuring better WiFi signal strength (5 dBi) at long range. The antenna can be rotated 180 degrees horizontally and 90 degrees vertically. It also supports WPA/WPA2 encryption and has universal compatibility with any router/gateway. This antenna is one of the most important components of the entire system, since it enables capturing probe requests sent by the devices.

According to the above-mentioned architecture, the Pi4 module can be configured in order to access the Internet via the LTE network through the USB connection with the 4G hat. Using Linux terminology, it is possible to associate the usb0 interface with the LTE communication module enabled for Internet navigation via the 1Nce Mobile Network Service directly from boot time. In our application, this functionality can be used to establish a communication channel with a remote service, e.g., a cloud platform, via TCP or HTTPS requests. The configuration of the 4G LTE module is based on a series of AT commands sent in a specific initialization script executed in the Pi4. Concerning WiFi communication, through the use of the external WiFi adapter, two separate network wireless interfaces are obtained, namely wlan0 corresponding to the internal network card of the Pi4 and wlan1 corresponding to the external adapter, both of which rest on the same subnet. By default, both interfaces are in managed mode, which means that the antenna will only be able to capture the packets with the MAC address of the device as their destination. To capture packets such as probe requests, the external antenna must be set to monitor mode. This way, wlan1 remains dedicated to packet capture, whereas wlan0 can still be used to connect to the Pi4 during development and testing, e.g., via ssh.

## 5. Unsupervised Clustering Algorithm on the Edge

In Section 3, we introduced probe request and MAC address randomization and presented some experiments carried out in order to extract information to be used to classify captured MAC addresses. To obtain a possible partitioning of collected MAC addresses, hence an estimation of the number of devices in the range of the scanner, we employ the DBSCAN (density-based spatial clustering of application with noise) algorithm, an unsupervised clustering algorithm used in machine learning and data mining. The name comes from the fact that it takes as input a set of points in a given space, clusters neighboring ones based on a given distance, and marks as noise those that are alone in low-density areas. This feature makes it very advantageous in that it does not need to specify a number of clusters a priori (i.e., unsupervised clustering) but must be derived using the notion of density that will be introduced later.

The DBSCAN algorithm [15] requires two parameters: minPts, which is the minimum number of points needed to form a cluster, as well as the threshold for considering a region of dense points, eps, which is a measure indicating the distance within which a point is considered part of the “neighborhood” of another point. These parameters can be understood if two density-related notions are introduced:

- *Reachability*: Point *p* is reachable from *q* if their distance is within the eps parameter.
- *Connectivity*: Points $p_{1}$ and $p_{n}$ are connected if there exist $p_{2}$, $p_{3}$... $p_{n-1}$ such that $p_{i+1}$ is reachable from $p_{i}$ for each i:1,…,n−1.

**Figure 5:**
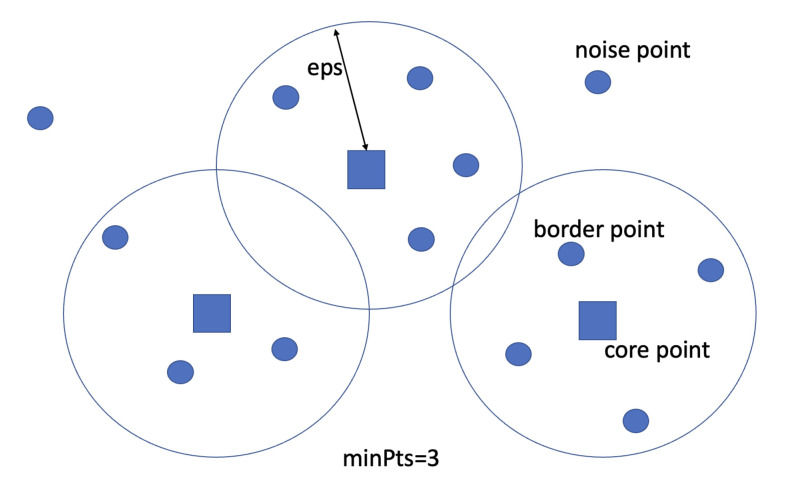
DBSCAN search space.

As shown in Figure 5, there are three types of points within the considered search space:

- *Core points* are the points having at least minPts points at distance eps;
- *Border points* are the points having at least one core point at distance eps;
- *Noise points* are neither core points nor border points.

The DBSCAN algorithm works as follows.

1. Given a set *S* of points, for every *P*, in *S*, repeat the following steps until all points have been visited.
  (1) If *P* has not been visited, we search for its set *N* of neighbors within the chosen eps distance and via a function indicating the type of distance to be computed distFunc. This step may require exploration of the entire dataset.
  (2) If the number of collected points in *N* is greater than or equal to minPts, then a new cluster *C* is created with them; otherwise, the analyzed point *P* is classified as a noise point.
  (3) For each point *Q* in the cluster *C* obtained in step (2), if it had been labeled as a noise point or was not visited, then it is given the label of the cluster obtained in that step as its label, and then, we expand the cluster recursively in the same way.

In the rest of the section, we describe a lightweight version of the DBSCAN algorithm, named DBSCAN${}_{L}$, customized on the types of data resulting from the preliminary analysis of the key elements of probe request frames analyzed in Section 3.

### 5.1. The Lightweight DBSCAN Algorithm

The DBSCAN${}_{L}$ algorithm is a simplified version of DBSCAN that partitions an initial address list according to two different features, namely RSSI and data rates, with an additional step based on other possible clustering criteria. Differently from the original version of the DBSCAN algorithm, in our algorithm, we do not merge overlapping clusters. The algorithm inserts in the current label only noise points, maintaining labels assigned in previous steps. In this paper, we focus on three possible variants of the algorithm, named standardCount, bruteforceCount and wordDistanceCount.

Every version of the algorithm uses the rangeQuery procedure defined in Algorithm 1 and takes in input two separate MAC address lists, one with the real addresses and the other with randomized addresses computed via a preliminary analysis of the address structure as the one discussed in Section 3. The latter list in particular must have the maximum RSSI with which the address was identified, as well as the list of supported data rates. In addition to these two lists, we consider three parameters: minPts, the RSSI-based eps parameters, and the weps parameters used in the wordDistanceCount algorithm.
**Algorithm 1** The rangeQuery procedure**Require:** *m*: address**Require:** *t*: metric type (rssi, dr, wd); eps: rssi range, weps: hamming range**Ensure:** rangeQuery returns the vicinity of address *m* according to the selected metrics.  **function**rangeQuery(m,t,M)    C=∅    **for** $m^{\prime }\in M$ **do**                **if** (D(t,m,m’)=true) **then**           $C=C\cup \{m^{\prime }\}$        **end if**    **end for**    return *C***end function** where $D\left(rssi,m,m^{\prime }\right)\;\equiv \;(|rssi\left(m\right)-rssi\left(m^{\prime })\right|\leq eps)$ $D\left(dr,m,m^{\prime }\right)\;\equiv \;\left(dr\left(m\right)=dr\left(m^{\prime }\right)\right)$$D\left(wd,m,m^{\prime }\right)\;\equiv \;\left(Hamming\_similarity\left(m,m^{\prime }\right)\geq weps\right)$

Given a set of addresses *M*, a partitioning is defined via a labeling mapping label that assigns cluster identifiers to elements in *M*. We assume here that cluster identifiers are natural numbers associated with the type of distance function, namely rssi,dr,wd, considered in the labeling algorithm. For instance, a cluster with label (rssi,1) can be partitioned in the subclusters with labels (dr,1) and (dr,2), and so on. Furthermore, we assume that addresses can also be labeled as noise or undef.

The three variants of the algorithm are defined on top of two functions, namely getCluster and clustering. More specifically, getCluster(i,t,M) denotes the cluster with label *i* (a number) obtained with distance of type *t* (e.g., dr,wd) from the set of addresses *M*. The function clustering described in Algorithm 2 computes a set of clusters created on the basis of the given distance type. The function rangeQuery in Algorithm 2 is used here to identify the set of elements in the proximity of a given element passed as the argument.
**Algorithm 2** The $DBSCAN_{L}$ algorithm in pseudo-code**Require:** *M*: set of addresses, label: mapping from *M* to address labels**Ensure:** label: updated with new cluster ids’ (numbers)  **function** clustering(t,M)    Id=0    **for** m∈M **do**                **if** label(m)∈{noise,undef} **then**           C=rangeQuery(m,t,M)           **if** |C|≤minPts **then**               label(m)=noise           **else**               Id=Id+1               label(m)=Id               **for** $m^{\prime }\in C$ **do**                   **if** $label\left(m^{\prime }\right)\in \left\{noise,undef\right\}$ **then**                       $label(m^{\prime })=Id$                   **end if**               **end for**           **end if**        **end if**    **end for**    return Id  **end function**

#### 5.1.1. First Variant: standardCount

The first version of the algorithm, namely the standardCount function in Algorithm 3, is defined as follows. In the first phase, given as input a list of randomized MAC addresses, the algorithm, via the function clustering, computes clusters created on the basis of the distances between RSSI signals. In the second phase, taking the set of clusters created in the first phase as input, an additional clustering step is carried out by using a distance defined as the equality of support data races. The estimated number of devices obtained from the randomized MAC addresses is then the number of subclusters that is formed upon termination of the second phase.
**Algorithm 3** The standardCOUNT procedure**Require:** real: set of real addresses;**Require:** fake: set of fake addresses;**Ensure:** the function returns an estimation of the number of devices.  **function** standardCount(real,fake)    N=cluster(rssi,fake)    fakeDevices=0    **for** k∈[1,…,N] **do**        C=getCluster(k,rssi,fake)        Id=clustering(dr,C)        fakeDevices←fakeDevices+Id    **end for**    return |real|+fakeDevices  **end function**

#### 5.1.2. Second Variant: standardCount

The first phase of the bruteForceCount variant of the algorithm, see Algorithm 4, is the same as that of standardCount. The second step however consists of a subcluster division operation based on what is the estimated number of randomized MAC addresses sent by a device per minute. In this version, it is required to specify both the duration of the time window in which the randomized MAC addresses were collected (win_size), as well as an estimated number of MAC addresses sent via probe requests from a device per minute (no_probes_per_device), which is normalized into the duration of the time window. Namely, for each identified subcluster, the following formula is applied:

(subcluster_length)/((no_probes_per_device)∗(win_size))

**Algorithm 4** The bruteForceCOUNT procedure**Require:** real: set of real addresses;**Require:** fake: set of fake addresses;**Ensure:** the function returns an estimation of the number of devices.  **function** bruteForceCount(real,fake)    N=cluster(rssi,fake)    fakeDevices=0    **for** k∈{1,N} **do**        C=getCluster(k,rssi,fake)        Id=clustering(dr,C)        fakeDevices←fakeDevices+Id        **for** p∈[1,…,Id] **do**           C=getCluster(p,dr,C)           estfake=round(|C|/(no_probes_as_device∗winSize))           **if** estfake≥2 **then**               fakeDevices←fakeDevices+estfake−1           **end if**        **end for**    **end for**    return |real|+fakeDevices**end function**

The estimated number of passengers is then the sum of the results obtained by applying the above formula, with rounding, to which the number of actual MAC addresses is added.

In this variant, we refine clusters that contain similar devices, either as signal strength or as support data rates, and find an easy way to handle crowded situations in transport means; no_probes_per_device should be derived experimentally.

#### 5.1.3. Third Variant: wordDistanceCount

In the wordDistanceCount variant, see Algorithm  5, we add an additional clustering phase on top of that obtained via standardCount. The refinement is based on the Hamming similarity of randomized MAC addresses. The parameter weps must be specified to create the clusters this time based on the number of equal symbols for each MAC address. The estimated number of passengers in this case is the sum of the number of real MAC addresses and the total number of Hamming-based subclusters identified.

We remark that, differently from the original DBSCAN algorithm, our clustering procedure does not merge overlapping clusters. The algorithm labels only noise points, maintaining labels assigned in previous steps. In our tests, the merge step produced a rough underestimation of the set of actual devices with reference to the adopted labeling strategy.

In the rest of the paper, we discuss experimental results obtained with the three variants of the algorithm implemented in the our edge device as explained in the next section.
**Algorithm 5** The wordDistanceCOUNT procedure.**Require:** real: set of real addresses;**Require:** fake: set of fake addresses;**Ensure:** the function returns an estimation of the number of devices.  **function** wordDistanceCount(real,fake)    N=cluster(rssi,fake)    fakeDevices=0    **for** k∈[1,…,N] **do**        C=getCluster(k,rssi,fake)        Id=clustering(dr,C)        fakeDevices←fakeDevices+Id        **for** p∈[1,…,Id] **do**           C=getCluster(p,dr,C)           WId=clustering(wd,C)           **if** WId≥2 **then**               fakeDevices←fakeDevices+WId−1           **end if**        **end for**    **end for**    return |real|+fakeDevices  **end function**

## 6. Edge Computing Pipeline Architecture

The software artifact used to control the Raspberry Pi and the 4G hat consists of the components executed periodically via the launch_apc script defined as follows:

sudo tcpdump -i wlan1 -e -s 0 -l type mgt subtype probe-req | \

python3 -u counter.py | python3 send_module.py

Multiprocessing is used to define the Python pipeline that implements the different steps required by the algorithm (probe sniffing, data preparation, estimate generation, and data distribution). The pipeline consists of the following components.

The tcpdump network traffic analysis tool remains listening on the AC650 antenna set in monitor mode on the wlan1 network interface, in order to capture all frames containing probe requests sent by devices in its vicinity. The data are printed on standard output with no buffering (option -l).

The counter Python script is the core of the pipeline. It takes as input the output produced by the tcpdump tool and performs two types of operations via separate threads.

- The first thread is in charge of preparing a dataset containing the collected MAC addresses, the timestamps corresponding to their first detection within the current epoch, the maximum RSSI with which it was detected, the list of date rates extracted from the probe request, and a classification tag distinguishing real and randomized addresses; the latter is also used to create two separate lists of MAC addresses. Filtering operations are also applied in this thread on the basis of the RSSI to avoid considering devices with low signals as potential passenger devices.
- The second thread processes the resulting data through one of the algorithms described in Section 5. The algorithm computes an estimate of the number of passengers and then produces as an output a json containing the estimate along with other data, including the number of real and randomized addresses found in the considered time window.

Threading is used here in order to apply the producer–consumer pattern using components working on the same address space, i.e., processing data in main memory only.

The send_module Python script manages LTE communication with the Senseioty cloud server via a custom API class flairbitAPI and collects GPS position via a dedicated script that control the GNSS receiver via AT commands. The component takes in input the json data produced by counter script and combines the crowd estimate with GPS data by updating a shared object kept in main memory. An additional task dumps the data to the remote server via a Rest invocation using an LTE-enabled Internet connection. The operations described above are all punctuated by a time window (epoch) in which collection takes place, construction of data lists, and at the end, estimation of passengers, cleaning of data lists and then starting again from collection. Inside the json, the headers section contains the name of the device assigned to the platform (serial), the timestamp for sending the data, and a payload section containing the GPS coordinates and the values of the measures obtained as a result of the send_module.py, which are, in addition to the timestamp:

- APC estimation: A string that represents a numerical range relative to the number of passengers, which can take the values 0–10, 10–25, 25–50 and more than 50, and is derived from the value of the APC-Counter.
- APC counter: the count obtained by the counter module by applying the algorithm.
  – REAL counter and FAKE counter: consist of the lengths of real and randomized MAC lists, respectively.
  – Used mode: the different modes introduced in Section 5. It can take the values MODE_0 (sum of real and randomized MAC addresses), MODE_1, MODE_2, MODE_3 (standardCount, bruteForceCount, wordDistanceCount, respectively).

**Figure 6:**
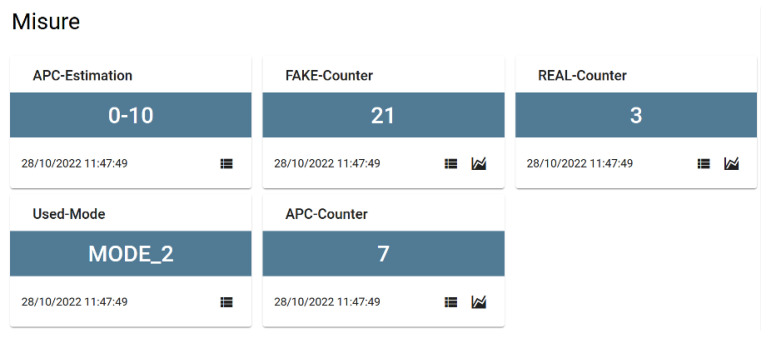
Dashboard.

Figure 6 illustrates the data displayed in the Senseioty dashboard [27]. Numerical values have two icons, the first of which is used to display the history of the data sent, which can be useful for further analysis, while the second is used to display some statistics on the data, such as the maximum or the daily average value. In addition, the names of the measures must coincide with those used in the scripts.

**Figure 7:**
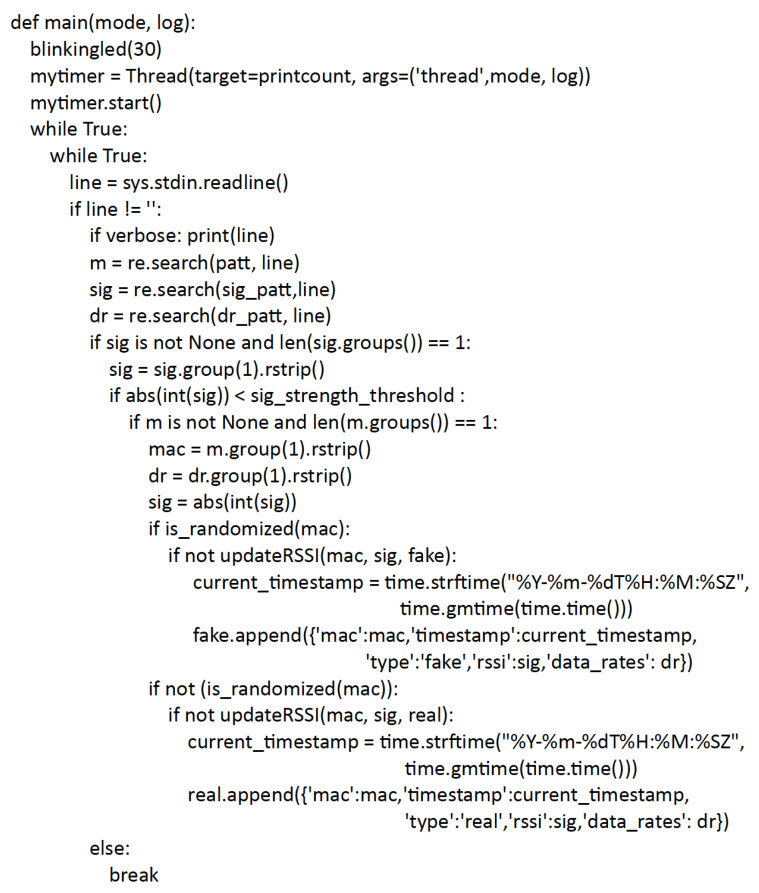
Counter script main loop.

Figure 7 shows the main part of the counter script including the part for processing the data captured by tcpdump, and for creating the dataset with the MAC addresses and related information.

**Figure 8:**
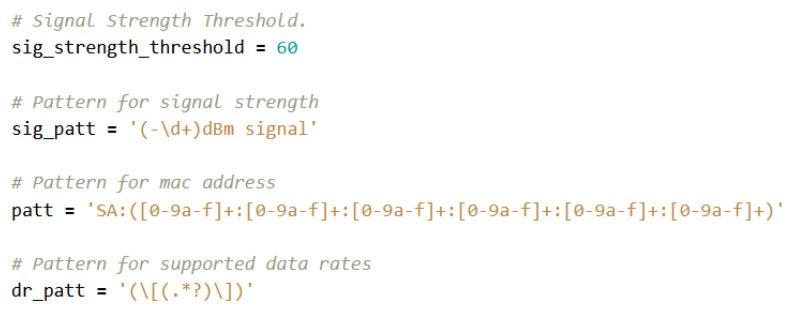
Threshold and regular expressions.

In each iteration of the loop of Figure 7 the script parses each row produced by tcpdump to extract three values, through the regular expressions illustrated in the Python code of Figure 8: the MAC address, the RSSI, and the list of supported data speeds. RSSI checks are also carried out, in which everything that is below a certain threshold (in this case –60 dBm) is discarded in order to avoid devices that are presumably out of the means of transport, and it is also converted to absolute value to be able to handle it more easily. After that, it is checked whether the MAC is randomized or not, and after a last possible check with RSSI update in case this is already present, the relative tuple is created in the dataset that will contain, respectively: MAC address, timestamp of first detection, classification in real or randomized, highest RSSI with which it was detected and list of supported data speeds. The data will take the following names, respectively: mac, timestamp, type, rssi, data rates.

**Figure 9:**
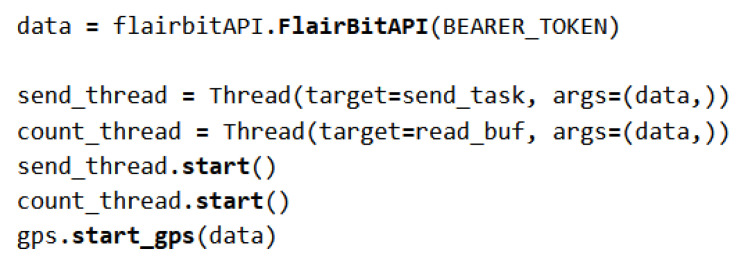
The send_module main part.

Figure 9 illustrate the main part of send_module. The flairbitAPI object is created to communicate with the Flairbit server, through which an access token provided by the company is passed. After that, two separate threads are started to which the created object is passed, one to update the fields with the data obtained from the counter (count_thread) and one for the actual sending of the data complete with GPS location.

**Figure 10:**
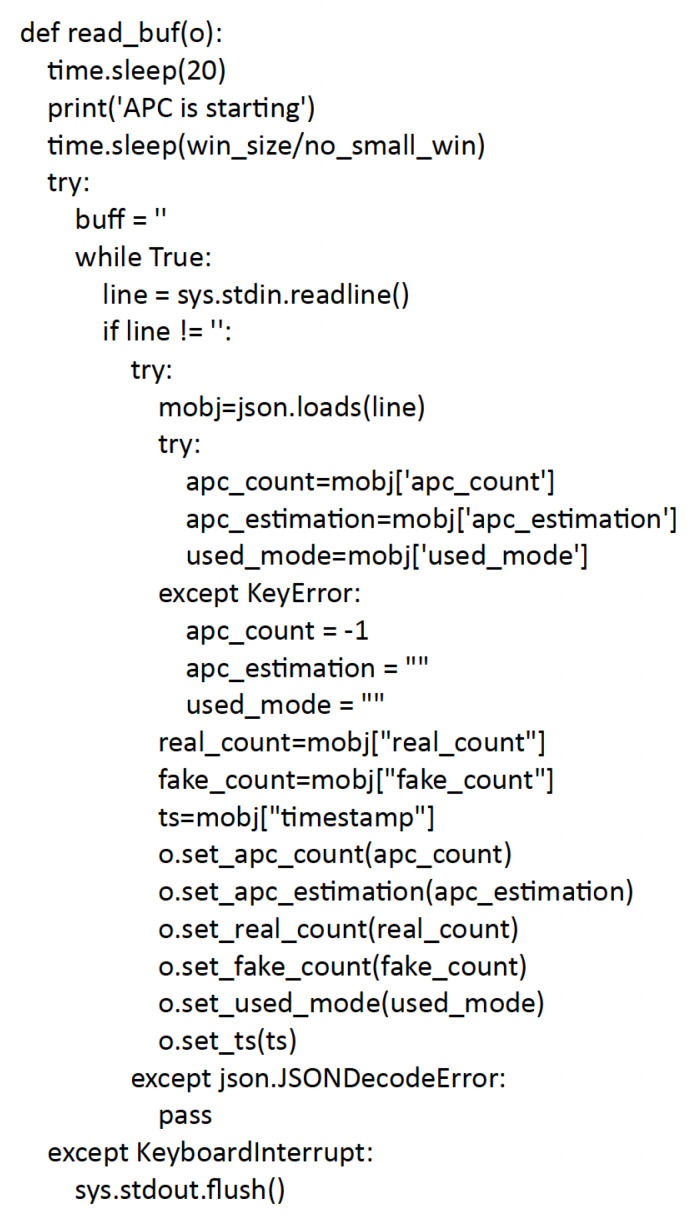
The read_buf procedure.

The read_buf thread, defined in Figure 10, takes the data sent from the counter module and updates the flairbitAPI object fields.

**Figure 11:**
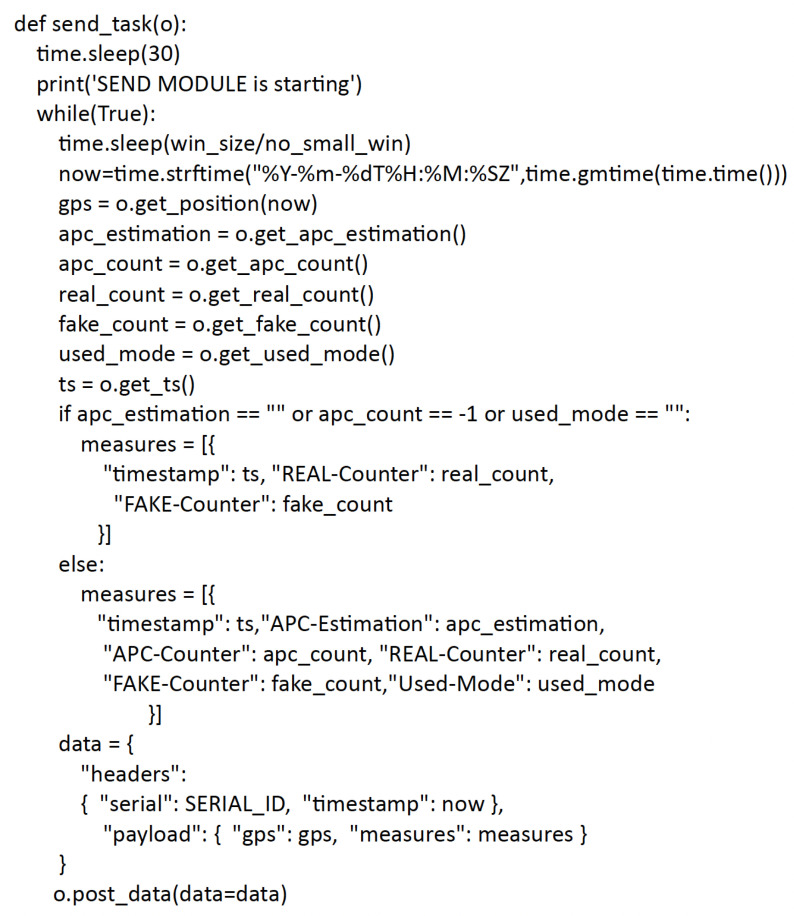
The send_task method.

Figure 11 illustrates the body of the thread, which is temporally synchronized with the sending of data from the counter script as indicated by the respective time sleep. It takes the data sent by the counter script from the input stream and then refreshes the flairbitAPI fields. The json message format used to send the data to the cloud server is shown in Figure 11. The send_task method builds the json message to be sent to Senseioty with data via POST method.

The epoch (time window) used for data acquisition is set in number of seconds, compatible with the functions to manage time sleep in Python, and it is possible, as already introduced previously, to divide the window into smaller ones, which is useful if for example there is a want to return an average of estimated values rather than just one (for example, for long-distance routes).

The artifact includes a simulation script, a program that takes into input two datasets of real and randomized MAC addresses in the form of .csv files and executes all three variants of the algorithm with different parameter values, and then returns, for each execution, the result obtained.

## 7. Experimental Evaluation

### 7.1. Motivations

Our tests were performed in different locations at our university campus with the aim of recreating different scenarios that may occur inside a bus and, in the meantime, to have more structure information on the set of devices used in the experiments. The tests are intended to learn thresholds to be used in the algorithms, as well as questioning choices made when creating the algorithms. More in detail, we considered the following scenarios.

### 7.2. Unsupervised Test

This test tries to simulate the bus so that we cannot know anything about the devices of the users. The goal here to determine if the estimated number of passengers obtained is similar to the counted people number in the test place. The test was carried out in a study place with around 30 or 35 students, all equipped with a smartphone/laptop. To capture WiFi probe signals, we positioned the antenna in one of the entrances to the area. We did not known whether or not both student devices were connected to the university WiFi network. The program discarded each frame with less than −60 dBm, since we assumed from previous experiments that it corresponds to the standard size of a bus. For real-time testing and data acquisition, we used the *bruteForceCount*, as it was considered the most promising solution, with the following parameters: *minPts = 2*, *rssiEps = 2* and *noProbesAsDevice = 4*. For *winSize*, we initially chose *120* as the value, which corresponds to two minutes, as we wanted to try to simulate a bus with frequent stops, but we tried to perform a manual merge to obtain windows of six minutes as well (*winSize = 360*) composed of three small windows, due to uncertainty related to the correct acquisition time and to the possibility of having datasets easier to analyze and manage. Six-minute datasets were composed by following the same code execution rules, so that for each MAC address, we had an RSSI with the highest value found, and the first list of data rates appeared.

The test lasted about an hour. Table 6 is related to the two-minute windows experiment. Looking at the *Estimated passengers number *column, the table shows a minimum of 9 passengers and a maximum of 23 passengers; thus, the range is very wide, as we start from high numbers at the beginning, and then, they decrease to lower and more stables values (except 22 in the observation 12:07:16). If we divide the table into three equal parts, the first has an average of 18.5 estimated passengers, while the last ones have an average of 15.4 and 15.3 passengers. This trend is consistent with the fact that at the beginning, there was a break time; thus, there were more students than the usual walking around with their smartphones. To confirm this, it is possible, for example, to observe the number of randomized MAC addresses: in the first part, there is an average of 44.4 MAC addresses, while in the last one an average of 32. In other words, there is some sensitivity to noise.

Table 7 is related to the six-minute windows experiment. Looking at the *Estimated passengers number* column, we have a range between 26 and 33 (excluding the peak of 20 at 11:50:35). If we split the table in two halves, we have an average of 31 passengers in the first half and 26,4 in the second half. Thus, we have now a more realistic and stable range, and we can confirm the size of the time window matters, as more data are being processed.

However, considering the coverage range concerns, the results from Table 6 seem more related to the first half of the study area, while the results from Table 7 seem more related to the study area in its entirety.

By increasing the *minPts* parameter to 4 and 8 in the six-minute dataset, we obtained the results shown in Table 8.

**Figure 12:**
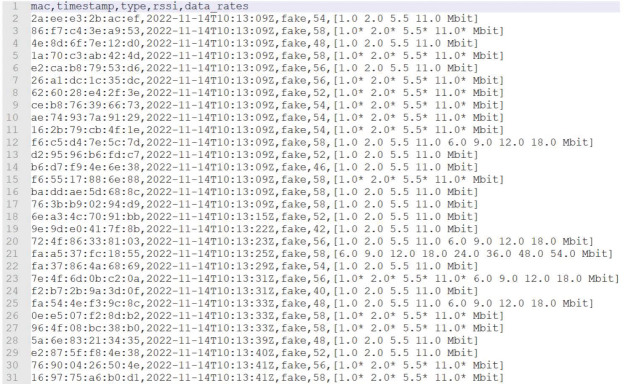
Example of acquired dataset from the unsupervised test with randomized MAC addresses.

According to Table 8, with *minPts = 4*, we have a range between 23 and 28 (excluding the peak of 17); thus, if we divide the results into two halves, we have an average of 25.8 for the first half and 23.2 for the second; thus, there is less difference, and it seems to be useful for reducing noise. However, the results are not similar to Table 6, even with *minPts = 8*, and the more that *minPts* increases, the more it may be necessary to increase the size of the time window, in order to not underestimate the results.It is not easy to identify the source of the noise. One cause is probably the presence of more people. In Figure 12, we can see that there are many MAC address captured with −58 dBm as the maximum RSSI, which is close to the threshold, and the possible explanation is that the study place was bigger than a bus. Thus, even if the students might seem close, they were not at all. In addition, the coverage range could be large; thus, maybe the farther the device is, the more likely it will be captured but with low signals (in our situation, from −50 to −59 dBm). Thus, it could potentially lead to noise. Fixing a value of *minPts* that depends on the observed RSSIs could be an interesting strategy to consider.

Table 7 seems to be more accurate than Table 6. Table 9 elaborates the data discussed thus far with the three different algorithms but while maintaining the value used for parameters in Table 7 (*rssiEps = 2*, *minPts = 2*).

We can see that *standardCount* and *bruteForceCount*, with its difference in parameters values, gave similar results, but it is still better to use the latter since it gives us extra precision due to the experiments related to the average number of MAC addresses sent by a device. In addition, with *noProbesAsDevice = 3*, we obtain a slightly higher value than *noProbesAsDevice = 4*.

Regarding *wordDistanceCount*, the result seems to be more uncertain, since with *wdEps* being smaller than 3, the value is too high, with *wdEps* equal to 4, we have a peak of 41; thus, it is quite reasonable but still too high, with *wdEps* being greater than or equal to 5, the results are more similar to *standardCount*. Thus, it is still difficult to understand if *wordDistanceCount* could be useful or not.

In conclusion, we can say that we have tested the algorithm in an environment of 35 people without having any control over the devices, and we have obtained the following outcomes:

- The algorithm (*bruteForceCount*) and parameters chosen at the beginning gave promising results, even if we had to “artificially” increase the size of the time window to obtain a satisfactory result
- We cannot confirm where the noise comes from, but we tried to analyze the dataset, and the solution could be to handle the coverage range differently or to increase the *minPts* parameter value to 4.

### 7.3. Supervised Test

The supervised test consisted of using a fixed number of devices in a room, with the aim of understanding whether the number of passengers is similar to the actual number of devices used for the test, as well as being able to control the devices, for example by turning the screen on or off.

As already mentioned, partial tests were conducted during the internship but only with very few devices; thus, we decided to conduct a similar test with a group of devices owned by the university, which were around 20 tablets used to simulate passengers, all of them produced by Lenovo with the exception of one. All the tablets were in the same condition as on a bus, thus with the WiFi turned on but not connected to any access point. Not all of the tablets worked; thus, the actual number of devices was 16 or 17, in which we added the two smartphones of the people that were in the test site, the two laptops and the Raspberry Pi. Thus, the total was around 21 or 22 active devices.

In this test, we faced the following obstacles, although it was an interesting “case study”: The test was conducted in the small office; thus, the tablets were not well spaced. However, it could be useful in order to test a crowded environment and specifically to verify if the algorithm is able to distinguish the devices Except for five devices, the tablets were all produced by Lenovo. Although it might be unrealistic for all users to have a device from the same producer, this scenario allowed us to check how the algorithm performs in a crowded place with some additional information coming from controlled devices.

The data were acquired for approximately 26 min, divided into two halves, with screens turned off and turned on. Time windows were again of two minutes, but the results with six minutes are displayed as well, since it could be used as confirmation that one of the two is more suitable. The algorithm and parameters were the same as for the unsupervised test, but the final results with the other configurations are provided as well.

Table 10 shows the results of the two-minute table and thus the results of the real-time test.

Table 11 shows the corresponding six-minute table.

In Table 11, we can confirm a better precision, since in the observation 14:33:34, we obtained 20 as the estimated passenger number, very close to the real device number. In addition, the number of real devices matches the number of devices we already knew would use their real MAC address, which were a smartphone, a laptop and the Raspberry Pi itself. Thus, according to the promise, the estimation is good.

However, there is a different balance if we divide both the tables into two halves. For example, if we take Table 11, both halves have an average of 3.5 real MAC addresses, but the first half has an average of 142 fake MAC addresses and the second one an average of 91.5.

This behavior is related to the fact that devices had screens turned off in the first half and screens turned on in the second half. This is different than what was stated in the experiments before the development of the algorithm, as most devices send more MAC addresses when the screen is turned on, and thus it may depend on the manufacturer.

**Figure 13:**
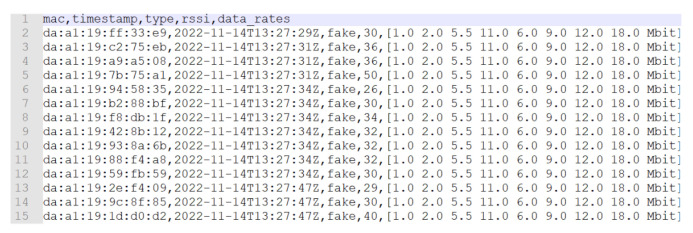
First example of acquired dataset from the supervised test with randomized MAC addresses.

**Figure 14:**
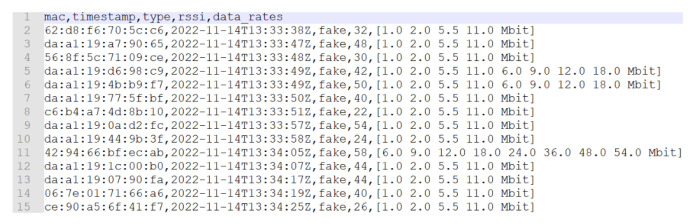
Second example of acquired dataset from the supervised test with randomized MAC addresses.

As shown in Figure 13 and Figure 14, many addresses with da:a1:19 as the prefix were found (which is used for randomized MAC addresses) and were often captured with a large RSSI range, from −26 to −50. This should prove that those addresses come from different devices in the room. Regarding the difference of the two pictures, the addresses of Figure 13 were captured in 18 s, while the addresses of Figure 14 were captured in 47 s; thus, it should be confirmed that not all devices send probes with screens turned on, and it depends more on the producer of the device. In addition, the devices sometimes send a different data rate list, but this is rare; thus, sub-clustering based on them is still very useful.

Table 12 shows the results from Table 11, with all three algorithms and the different parameter values. We can see that the *wordDistanceCount* has a different result compared to the unsupervised test, as the results are more similar to those of the other two algorithms, because the MAC addresses of Lenovo tablets all have the same prefix. Thus, the algorithm struggles to create clusters based on word distance, where, at this point, the *wordDistanceCount* is quite unstable, at least with this implementation.

In conclusion, we can say that we tested the algorithm with a set of controlled devices, all in the conditions to send probes, but with difficulties related to the same producer and the considered environment. However, we inferred the following considerations: the choice of algorithm and parameter values used can be confirmed to be good in the considered experiments; not all devices behave the same when sending probe requests; some types of device send probes only when the screen is turned off.

## 8. Conclusions

In this paper, we presented a solution for computing crowd density estimations via WiFi scanning and AI algorithms deployed on edge devices in order to ensure data privacy. The prototype was tested in supervised and unsupervised testing. Software architecture was implemented in order to facilitate possible extensions.

Although inspired by AI algorithms such as those applied in [4,5,6,10], one distinguished feature of our approach was the use of a hierarchical clustering algorithm that adjusts clusters via a sort of incremental refinement procedure. Another interesting feature of our approach is that the processing pipeline is highly modular. Furthermore, the entire procedure was implemented on the edge for preserving data privacy and was tested and executed on a custom device built on top of low-cost components. We also differentiated the testing phase by considering both supervised and unsupervised scenarios to compare the results, e.g., with a fleet of known a priori or unknown devices.

A combination of different clustering methods, using for instance a consensus strategy or ensemble clustering techniques to combine multiple clustering results into a more robust clustering, is a possible future direction for extending our approach and software artifact. For instance, in our experiments, we did not consider sequence numbers as in [19], and we applied instead other types of similarity metrics. Additional metrics could be particularly useful when combining different sniffing tools. 

## Figures and Tables

**Table 1:** Example of RSSI data extracted via tcpdump.

08:22:06.305161 −92 dBm signal SA:3e:c1:b7:1e:dc:38	08:22:07.335281 −18 dBm signal SA:3e:c1:b7:1e:dc:38
08:22:06.567528 −38 dBm signal SA:3e:c1:b7:1e:dc:38	08:22:07.372982 −18 dBm signal SA:3e:c1:b7:1e:dc:38
08:22:06.640095 −38 dBm signal SA:3e:c1:b7:1e:dc:38	08:22:07.660441 −38 dBm signal SA:3e:c1:b7:1e:dc:38
08:22:06.876389 −18 dBm signal SA:3e:c1:b7:1e:dc:38	08:22:07.699966 −38 dBm signal SA:3e:c1:b7:1e:dc:38
08:22:06.914380 −18 dBm signal SA:3e:c1:b7:1e:dc:38	08:22:08.027113 −54 dBm signal SA:3e:c1:b7:1e:dc:38
08:22:06.952107 −18 dBm signal SA:3e:c1:b7:1e:dc:38	08:22:08.064867 −56 dBm signal SA:3e:c1:b7:1e:dc:38
08:22:07.031088 −16 dBm signal SA:3e:c1:b7:1e:dc:38	08:22:08.102936 −54 dBm signal SA:3e:c1:b7:1e:dc:38
08:22:07.068810 −18 dBm signal SA:3e:c1:b7:1e:dc:38	08:22:08.181699 −58 dBm signal SA:3e:c1:b7:1e:dc:38
08:22:07.106561 −16 dBm signal SA:3e:c1:b7:1e:dc:38	08:22:08.222457 −60 dBm signal SA:3e:c1:b7:1e:dc:38
08:22:07.297354 −18 dBm signal SA:3e:c1:b7:1e:dc:38	08:22:08.260062 −56 dBm signal SA:3e:c1:b7:1e:dc:38

**Table 2:** RSSI data extracted via tcpdump, with distances smaller than 1 m.

12:57:00.305354 −28 dBm signal SA:52:b2:f8:04:50:bf
12:57:22.066625 −30 dBm signal SA:02:a0:dc:7c:05:aa
12:57:30.671233 −28 dBm signal SA:be:37:03:70:9e:5d
12:57:47.670341 −30 dBm signal SA:3a:0c:28:c9:6d:24
12:57:59.750892 −32 dBm signal SA:86:10:0c:f5:8e:96

**Table 3:** RSSI data extracted via tcpdump, distance greater than 4 m.

13:44:37.113393 −44 dBm signal SA:16:60:64:de:4c:76
13:44:47.656279 −32 dBm signal SA:b6:02:ef:16:d1:ee
13:44:52.950555 −36 dBm signal SA:9e:c9:07:ad:72:d8
13:45:04.041955 −36 dBm signal SA:42:6b:e4:8f:ff:00
13:45:26.143811 −38 dBm signal SA:56:5c:cd:5f:0a:04

**Table 4:** MAC addresses from Galaxy M12.

3e:c1:b7:1e:dc:38	a6:e1:2c:e0:26:04
5e:5d:b1:f4:80:50	ae:14:f1:ba:70:a4
5e:3a:57:af:23:81	16:a8:b1:d3:89:d7
1e:d5:49:00:c5:2d	3a:4d:61:50:c7:87
ea:97:3b:5b:15:0b	52:29:27:e5:04:4b
8e:db:07:d9:79:f6	c6:fd:ec:9c:3c:6a
02:f6:ee:10:de:25	22:f9:97:c1:ca:a1
d2:9e:17:ab:6c:1d	f6:45:66:5f:d2:bf
	aa:11:3d:97:97:14

**Table 5:** MAC addresses from Galaxy M12.

1	5e:5d:b1:f4:80:50	ae:14:f1:ba:70:a4	16:a8:b1:d3:89:d7
2	a6:e1:2c:e0:26:04	aa:11:3d:97:97:14	
3	ae:14:f1:ba:70:a4	5e:5d:b1:f4:80:50	aa:11:3d:97:97:14
4	16:a8:b1:d3:89:d7	5e:5d:b1:f4:80:50	
5	3a:4d:61:50:c7:87	f6:45:66:5f:d2:bf	
6	52:29:27:e5:04:4b	22:f9:97:c1:ca:a1	
7	22:f9:97:c1:ca:a1	52:29:27:e5:04:4b	
8	f6:45:66:5f:d2:bf	3a:4d:61:50:c7:87	
9	aa:11:3d:97:97:14	a6:e1:2c:e0:26:04	ae:14:f1:ba:70:a4

**Table 6:** Unsupervised test results with original two-minute time window.

Timestamp	Estimated Passengers Number	Processed Real MAC Addresses	Processed Randomized MAC Addresses
11:15:09	21	12	62
11:17:14	23	15	44
11:19:19	16	8	42
11:21:24	15	10	30
11:23:29	18	11	30
11:25:34	21	13	39
11:27:39	16	10	39
11:29:45	19	10	65
11:31:50	21	12	56
11:33:55	15	10	37
11:36:00	21	12	39
11:38:05	21	11	47
11:40:10	20	8	64
11:42:15	17	7	60
11:44:20	14	8	29
11:46:25	10	6	21
11:48:30	9	5	27
11:50:35	11	6	27
11:52:40	15	9	24
11:54:45	15	9	21
11:56:50	15	9	27
11:58:56	14	9	23
12:01:01	14	9	32
12:03:06	14	7	45
12:05:11	15	8	35
12:07:16	22	14	32
12:09:21	15	7	30
12:11:26	15	7	28
12:13:31	13	5	43
12:15:36	17	13	25

**Table 7:** Unsupervised test results with adapted six-minute time window.

Timestamp	Estimated Passengers Number	Processed Real MAC Addresses	Processed Randomized MAC Addresses
11:19:19	32	21	146
11:25:34	31	19	92
11:31:50	29	18	158
11:38:05	30	18	118
11:44:20	33	15	150
11:50:35	20	11	73
11:56:50	26	17	69
12:03:06	27	17	96
12:09:21	31	21	90
12:15:36	28	14	106

**Table 8:** Results of Table 2 with different values for *minPts*.

Timestamp	Estimated Passengers Number with minPts = 4	Estimated Passengers Number with minPts = 8
11:19:19	28	27
11:25:34	26	24
11:31:50	25	24
11:38:05	24	23
11:44:20	26	22
11:50:35	17	14
11:56:50	25	21
12:03:06	23	22
12:09:21	28	27
12:15:36	23	20

**Table 9:** Results of Table 2 with different algorithms and values for parameters.

Timestamp	*standardCount*	*bruteForceCount*, *noProbesAsDevice* *= 3,4,5*	*wordDistanceCount*, *wdEps = 3,4,5*
11:19:19	32	33, 32, 32	65, 36, 32
11:25:34	31	31, 31, 31	38, 31, 31
11:31:50	28	31, 29, 28	82, 41, 28
11:38:05	29	30, 30, 29	61, 41, 29
11:44:20	32	33, 33, 32	64, 40, 32
11:50:35	20	20, 20, 20	34, 21, 20
11:56:50	26	26, 26, 26	29, 26, 26
12:03:06	27	27, 27, 27	43, 30, 27
12:09:21	31	31, 31, 31	46, 33, 31
12:15:36	28	28, 28, 28	39, 28, 28

**Table 10:** Supervised test results with original two-minute time window.

Timestamp	Estimated Passengers Number	Processed Real MAC Addresses	Processed Randomized MAC Addresses
14:23:09	10	2	36
14:25:14	9	3	40
14:27:19	10	3	33
14:29:24	12	2	65
14:31:29	13	3	46
14:33:34	12	2	54
14:35:39	8	2	30
14:37:44	8	3	16
14:39:49	4	3	6
14:41:55	8	4	17
14:44:00	12	4	38
14:46:05	6	1	25

**Table 11:** Supervised test results with adapted six-minute time window.

Timestamp	Estimated Passengers Number	Processed Real MAC Addresses	Processed Randomized MAC Addresses
14:27:19	14	4	119
14:33:34	20	3	165
14:39:49	13	3	52
14:46:05	17	4	79

**Table 12:** Results of Table 6 with different algorithms and values for parameters.

H,M,S Observation	Estimated Passengers Number with *standardCount*	Estimated Passengers Number with *bruteForceCount* and *noProbesAsDevice* * = 3,4,5*	Estimated Passengers Number with *wordDistanceCount* and *wdEps = 3,4,5*
14:27:19	13	15, 14, 13	13, 13, 13
14:33:34	19	21, 20, 19	23, 21, 19
14:39:49	13	13, 13, 13	18, 13, 13
14:46:05	17	17, 17, 17	19, 17, 17

## Data Availability

Data can be made available to interested readers by contacting the authors.
